# Validity and reliability of the Turkish version of the Michigan Neuropathy Screening Instrument

**DOI:** 10.3906/sag-1906-63

**Published:** 2020-06-23

**Authors:** Duygu AKTAR REYHANİOĞLU, Süleyman Cem ADIYAMAN, Murat BEKTAŞ, Onur BULUT, Başak ÖZGEN SAYDAM, Fırat BAYRAKTAR, Bilge KARA

**Affiliations:** 1 Dokuz Eylül University, Graduate School of Health Sciences, Department of Physical Therapy and Rehabilitation, İzmir, Turkey; 2 Dokuz Eylül University, Faculty of Medicine, Department of Endocrinology and Metabolism, İzmir Turkey; 3 Dokuz Eylül University, Faculty of Nursing, İzmir Turkey; 4 Dokuz Eylül University, Faculty of Medicine, Department of Neurology, İzmir Turkey; 5 Dokuz Eylül University, Department of Physical Therapy and Rehabilitation, İzmir Turkey

**Keywords:** Michigan, diabetic neuropathy, diabetes mellitus, reproducibility of results

## Abstract

**Background/aim:**

The aim of this study was to demonstrate the validity and reliability of the Turkish version of the Michigan Neuropathy Screening Instrument (MNSI-TR).

**Materials and methods:**

The study included 127 patients aged 45–76 years who were previously diagnosed with type 1 or 2 diabetes. Stability of the instrument was assessed by intraclass correlation coefficient. Reliability of the MNSI-TR was assessed using the Kuder–Richardson formula 20 test, item-total correlations, and floor/ceiling effect. Validity was evaluated with receiver operating characteristic curve analysis. A logistic regression model was used to determine to what degree the MNSI-TR explain nerve conduction study (NCS) results in the prediction of neuropathy.

**Results:**

With a cut-off value of 3.5 for the questionnaire, sensitivity and specificity of the MNSI-TR were 75.5% and 68.1%, respectively. A cut-off of 2.75 for the physical assessment part of the scale resulted in 87.5% sensitivity and 93.6% specificity. The scale was able to diagnose neuropathy in the rate of 71.5% of the patients diagnosed with neuropathy by NCS.

**Conclusion:**

The MNSI-TR is a valid and reliable method for evaluating diabetic peripheral neuropathy in Turkish speaking societies. It must be obtained a minimum of 4 points from the questionnaire part and a minimum of 2.5 points from the physical assessment part for the diagnosis of neuropathy

## 1. Introduction

World Health Organization data indicate that diabetes mellitus (DM) is one of the fastest growing health problems worldwide and has several complications that threaten human health [1]. Peripheral neuropathy is a common complication of DM, affecting nearly 60% of diabetes patients worldwide [2].

Sensory symptoms such as pain, burning, pinching, tingling, and numbness are common in diabetic peripheral neuropathy [3–5]. Other important clinical signs seen in people with neuropathy are loss of vibration sense, decreased touch, and proprioceptive sensation in the lower extremities, disappearance of the ankle reflex, and foot deformities, ulcerations, and amputations [2,6,7]. Therefore, early diagnosis is vital for preventing the progression of diabetic neuropathy [5].

Nerve conduction studies are considered the gold standard for neuropathy diagnosis but results are usually normal in pure small fiber neuropathy if the thick fibers are not affected. Another alternative is skin biopsy. However, it is not usually the first choice for neuropathy due to the difficulties in performing and interpreting the results [8–12]. Therefore, electrophysiological testing is considered the preferred technique [13].

Feldman et al. developed the Michigan Neuropathy Screening Instrument (MNSI) to facilitate the detection of neuropathy [14]. This low-cost and rapid tool was validated in numerous studies analyzing its reliability and precision [14–17]. The MNSI has two sections: section A consists of 15 self-administered questions about neuropathic symptoms, while section B includes a lower-extremity examination and assessment of vibration sense, ulceration, and ankle reflexes conducted by a clinician [14].

The MNSI has been adapted into many languages such as Portuguese and Brazilian Portuguese [18,19]. A valid and reliable Turkish version of the MNSI will help standardize the assessment of neuropathic patients in Turkey. Therefore, we conducted this study to determine the validity and reliability of the Turkish version of the MNSI (MNSI-TR).

## Materials and methods

This study was conducted in collaboration by the departments of neurology, endocrinology, and physical therapy and rehabilitation of Dokuz Eylül University Hospital. The study was approved by the Dokuz Eylül University ethics committee (approval no: 2018/25-27). In accordance with the Declaration of Helsinki, all patients provided written informed consent before the study.

The study group included patients 45 to 76 years of age with type 1 or 2 DM according to the American Diabetes Association (ADA) criteria [20] who presented to our hospital between August and December 2018 and were referred for nerve conduction study (NCS). Participants with alcoholism, chronic kidney and liver failure, history of cerebrovascular disease, cancer, chemotherapy and/or radiotherapy exposure, autoimmune disease, chronic infectious diseases, drug abuse, radicular neuropathy, and mental or physical disabilities were excluded from the study due to the increased risk of nondiabetes-related neuropathy. Patients who were blind or did not speak Turkish were also excluded. Illiterate patients were asked to administer the MNSI questionnaire with a literate relative. Antidepressant and anticonvulsant usage were not considered exclusion criteria. All participants underwent examination by both a neurologist and an endocrinologist and their history, sociodemographic data, body mass index, education level, type and duration of diabetes, and physical examination and laboratory findings such as fasting blood glucose and HbA1c were recorded. 

A clinical neurophysiology fellow performed nerve conduction study for all patients. In the upper extremity, sensory and motor potentials of the unilateral median and ulnar nerves, for the lower extremity, unilateral peroneal and tibial motor nerves and sural sensory nerve were studied. In each extremity on the opposite side, one motor and one sensory nerve potential was also evaluated for excluding assymetric polyneuropathy and plexopathy. Differences in amplitude, conduction velocity, and/or F-wave latency in at least two different nerves were required for a diagnosis of peripheral neuropathy. In addition to neurological examination findings, abnormality of amplytude, conduction velocity reduction and/or F-wave latency prolongation that is symmetric, length dependent and could not reach the demyelinating range was considered as distal symmetric neuropathy [21]. The physical examination part of the instrument was implemented by a physiotherapist with master’s degree who had no idea about the existence of neuropathy.

### 2.1. Application of the Michigan Neuropathy Screening Instrument

The MNSI has two sections, a history questionnaire completed by the patient (section A) and a physical assessment conducted by a medical professional (section B).

Section A assesses clinical symptoms via 15 yes/no questions. All questions except 7 and 13 are scored as 1 point if answered ‘yes’; questions 7 and 13 are scored as 1 point if answered ‘no’. Question 15 is evaluated by the clinician. Because question 4 can relate to impaired circulation and question 10 to general asthenia, neither was included in the original MNSI scoring [14]. A cut-off point of 7 for section A was initially accepted as abnormal; however, later studies showed that adjusting the cut-off point to 4 improved the performance of the MNSI [17].

Section B involves a clinical assessment of the feet including examination for dry skin, calluses, infections, fissures, and ulcers, followed by evaluation of ankle reflexes and vibration perception. Each foot with a deformity counts as 1 point and each foot with an ulcer also counts as 1 point. Ankle reflex is scored as 0 if present, 0.5 points if present only when the patient performs Jendrassik maneuver, and 1 point if absent despite the Jendrassik maneuver. Vibration perception is assessed using a 128-Hz tuning fork at the distal interphalangeal joint of the participants’ great toe. The score is determined based on how much longer the examiner can feel the vibration than the patient. If the examiner feels the vibration for 10 or more seconds longer than the patient, vibration sense is considered decreased and scored as 0.5 points. If the examiner feels the vibration for less than 10 s longer, vibration sense is considered normal (0 points). If the patient cannot sense the vibration at all, they receive 1 point. The maximum possible score from section B is 8 points, and a score greater than 2 is considered abnormal according to the original scoring algorithm [14].

Prior to the study, we obtained permission from Dr. Eva Feldman (first author of the original MNSI) to adapt the instrument to Turkish. Translation and cross-cultural adaptation of the MNSI were conducted as recommended by Bouton et al. [22].

First, two bilingual translators (native Turkish with English as a second language) independently translated the original version of the MNSI from English to Turkish. The original MNSI was compared independently with both of the translations and a common translation was prepared based on feedback from specialists. Finally, two native English-speaking translators with Turkish as a second language and no knowledge of the original MNSI back-translated the instrument from Turkish to English. The translators and health professionals agreed on a revised Turkish version, which was pretested with 19 patients. The participants provided feedback and any mismatches between the original and Turkish version were identified and reviewed.

The adaptation process concluded by presenting all documents to the language expert and content validity committee, and the most plausible translation was obtained.

### 2.3. Content validity

A team of seven experts evaluated the Turkish version of the instrument. The team compared the original and translated versions and scored the appropriateness of each item between 1 and 4 points (1 = requires substantial editing, 2 = requires minor editing, 3 = good, 4 = excellent). The items were changed based on these recommendations. Content validity indexes were calculated for both item-level (I-CVI) and scale-level (S-CVI). The number of experts who scored an item either 3 or 4 (thus dichotomizing the ordinal scale into relevant and irrelevant) was summed and divided by the total number of experts. S-CVI was calculated by dividing the sum of the I-CVI values by the number of items. Concordance of the expert opinions is expected to be greater than 80% [23,24].

### 2.4. Sample size

Minimum sample size was calculated based on Hatcher’s 100 rule (1994), which states that the sample size must be at least 100 people or 5 times the number of questionnaire items (15 items × 5 = 75) [25]. We initially evaluated 170 people; however, after excluding patients because NCS or physical assessment could not be completed or they were lost to follow-up, the final number of evaluated participants was 127.

### 2.5. Statistical analysis

Data were analyzed using SPSS for Windows version 22.0. Descriptive statistics were analyzed using percentage, mean, and standard deviation (SD). Test-retest reliability analysis was conducted by paired-samples t-test and Pearson correlation analysis. The retest was done three weeks after the pretest with 19 participants [26]. The desired correlation coefficient between the first and second tests is ≥0.70 [27,28].Internal consistency of the questionnaire was analyzed using the Kuder–Richardson Formula 20 (KR-20) coefficient. A KR-20 coefficient between 0.80 and 1.00 indicates excellent reliability, while a value between 0.60 and 0.80 is considered highly reliable [25,26]. Stability of the instrument was assessed by intraclass correlation coefficient (ICC). Pearson correlation analysis was used to analyze the relationship between item-total and item-subscale total scoring. It shows the similarity or invariance of measurements obtained by ICC at the same or different times from individuals. ICC above 0.60 is considered a good fit. According to the literature, the correlation coefficient between item-total score and item-subscale total score should be at least 0.20 and floor and ceiling effects should be under 0.15 to consider an instrument homogeneous [27].Determining floor and ceiling effects are also recommended as an indicator of scale reliability and validity. Minimum score obtained from the scale gives the floor effect while the maximum score gives the ceiling effect. If the proportion of respondents receiving floor and ceiling scores exceeds 15%, the scale may pose a problem both in reliability and validity [27].Validity was assessed with receiver operating characteristic (ROC) curve analyses. Based on the area under the ROC curve (AUC) values, the discriminating power of the instrument was interpreted as excellent at AUC >0.91, very good at 0.81–0.90, acceptable at 0.71–0.80, statistically nonsignificant at 0.50–0.70, and nondiscriminatory at <0.5 [29]. The optimal cut-off points for the two sections of the instrument were determined using a diagnostic index and Youden’s index, the score corresponding to the point where these two indexes are highest is determined as the cut-off point for that scale, and the sensitivity and specificity values were determined for those cut-off points [30].Logistic regression analysis was performed to determine the agreement between the number of patients diagnosed with neuropathy as a result of NCS and the number of patients diagnosed with neuropathy by scale.

## 3. Results

The study included 127 participants. Of these, 112 (88.2%) had type 2 DM and 15 (11.8%) had type 1 DM. The mean duration of diabetes was 11.42 (SD 7.47) years and the mean age of the participants was 59.77 (SD 10.02) years. Eighty-two (64.6%) of the patients were woman and 45 (35.4%) were men. Mean BMI was 29.11 (SD 5.07) kg/m², HbAlc value was 7.70% (SD 1.53%), fasting blood glucose was 153.82 (SD 58.11) mg/dL. Level of education completed by the participants was high school for 17.5%, middle school for 28.3%, and elementary school for 42.5%, while the remaining 11.8% of the patients were uneducated.

### 3.1. Content validity

Agreement between the experts ranged between 0.85 and 1 for each item (I-CVI) and was 0.95 for the entire scale (S-CVI).

### 3.2. Reliability analysis for section A

Test-retest analysis of questionnaire was shown in Table 1. There was no significant difference between the first application score and the second application score of the scale (P > 0.05). The correlation between the two measurements was found to be 0.977 (Table 1). Reliability analysis of questionnaire was shown in Table 2. The Kuder–Richardson reliability coefficient for Part A was found to be 0.732. The ICC was determined to be 0.649 for section A. There was no floor or ceiling effect for section A (Table 2). Item-total and item-subscale total scores of correlations were 0.341–0.646 (Table 3).

**Table 1:** Test–retest analysis of the MNSI (n = 19).

	n	Mean(SD)	z	P	r*	P
Test	19	6.26 (2.07)	1.000	0.317	0.977	0.000
Retest	19	6.15(2.14)				

r*: correlation coefficient

**Table 2:** Reliability analyses of the sections A and B (n = 127).

Item-subscale	Kuder–Richardson α	ICC*	Mean (SD)	Floor effect%	Ceiling effect%
Section A total	0.732	0.649	4.39 (2.60)	10.2	0.0
Section B total	0.604	0.439	3.18 (1.78)	5.5	0.8

*ICC: Intraclass correlation coefficient

**Table 3:** Item-total scores correlation coefficient for Section A (n = 127).

Items	Item-total score correlation (r)*
1. Are your legs and/or feet numb?	0.629*
2. Do you ever have any burning pain in your legs and/or feet?	0.570*
3. Are your feet too sensitive to touch?	0.533*
4. Do you get muscle cramps in your legs and/or feet?	0.341*
5. Do you ever have any prickling feelings in your legs or feet?	0.606*
6. Does it hurt when the bed covers touch your skin?	0.450*
7. When you get into the tub or shower, are you able to tell the hot water from the cold water?	0.004
8. Have you ever had an open sore on your foot?	0.345*
9. Has your doctor ever told you that you have diabetic neuropathy?	0.521*
10. Do you feel weak all over most of the time?	0.455*
11. Are your symptoms worse at night?	0.646*
12. Do your legs hurt when you walk?	0.504*
13. Are you able to sense your feet when you walk?	0.013
14. Is the skin on your feet so dry that it cracks open?	0.492*
15. Have you ever had an amputation?	0.122

r*: correlation coefficient

### 3.3. Validity analysis for section A

**Figure 1:**
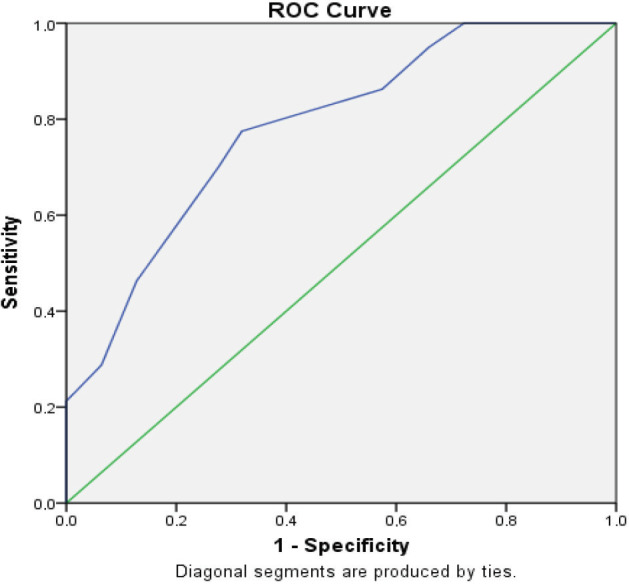
Determination of the cut-off point for section A according to ROC curve analysis.

A cut-off point of 3.5 was identified in ROC analysis according to the diagnostic and Youden’s indexes (Figure 1) The AUC was 0.783 and sensitivity found as 75.5% while specificity found as 68.1% for section A (Table 4).

**Table 4:** Cut-off point, estimation values, and area under the curve (AUC) values for prediction of neuropathy according to the ROC Analysis for sections A and B.

	Cut-off point	Sensitivity	Specificity	P	AUC* (%95 CI)	Diagnostic Index	Youden’s Index
Section A	3.5	0.755	0.681	0.000	0.783	1.456	0.456
Section B	2.75	0.875	0.936	0.000	0.939	1.811	0.811

*Area under curve

### 3.4. Reliability analysis for section B

Reliability analysis results was shown in Table 2. The Kuder–Richardson reliability coefficient for section B was found to be 0.604. The ICC was determined to be and there was no floor or ceiling effect for section B (Table 2). Correlations of item-total and item-subscale scores were between 0.372 and 0.757 (Table 5).

**Table 5:** Item-subscale total scores correlation coefficient for section B.

Items	Item-total score correlation (r)
Appearance of feet	0.680*
Ulceration	0.372*
Ankle reflexes	0.757*
Vibration	0.655*

r: correlation coefficient

### 3.5. Validity analysis for section B

**Figure 2:**
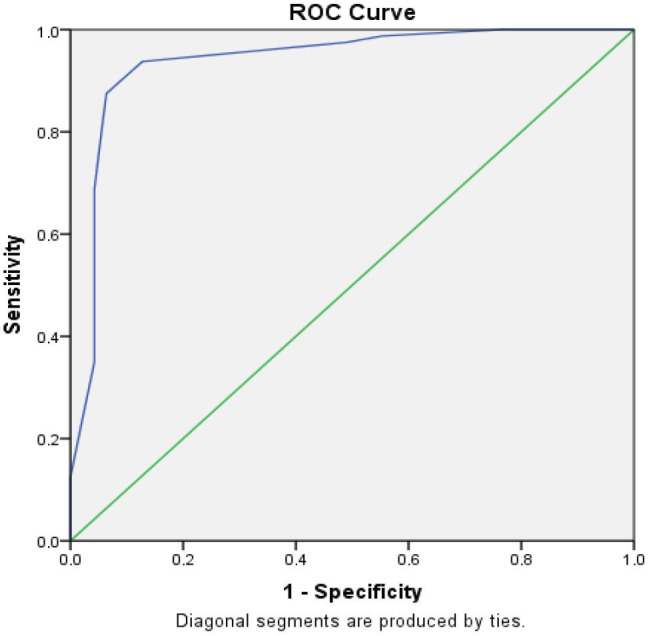
Determination of the cut-off point for section B according to ROC curve analysis.

The cut-off point for section B was 2.75 (according to the diagnostic and Youden’s indexes) and the AUC was 0.939 (Figure 2). For section B sensitivity was found as 87.5% and specificity was 93.6% (Table 4). We detected a moderate positive correlation between sections A and B (r = 0.519, P = 0.00). Item-subscale total scores correlation coefficient for Section B were between 0.372 and 0.757 (Table 5).Logistic regression analysis was performed to determine the agreement between the number of patients diagnosed with neuropathy as a result of NCS and the number of patients diagnosed with neuropathy by scale. 71.5% of the patients diagnosed neuropathy by NCS were diagnosed neuropathy by the two sections of the MNSI-TR. Increasing the score of section B indicates that the risk of neuropathy increases 5.526 fold (β = 5.526), and increasing the score of section A indicates that the risk of neuropathy increases 1.245 fold (β = 1.245) (Table 6).

**Table 6:** Association between sections of the instrument and NCS results (Logistic regression analyses).

Variables							95% CI
	B	SE	Wald	Df	Sig	Exp (B)	Lower	Upper
Section A	0.219	0.134	2.682	1	0.101	1.245	0.958	1.619
Section B	1.709	0.345	24.577	1	0.000	5.526	2.811	10.869
-2Log likelihood	73.185							
Cox & Snell R Square	0.524							
Nagelkerke R Square	0.715							

CI: Confidence interval, B: Beta, SE: Standard Error, Df: Degree of freedom, Sig: Significance, (B): Standardized Beta

## 4. Discussion

In this study, we evaluated both the validity and reliability of a Turkish version of the MNSI. We found MNSI-TR is a reliable and valiable form with a cut-off value of 3.5 and sensitivity and were 75.5% and 68.1%, respectively for the questionnaire part and for the physical assessment part of the scale resulted sensitivity of 87.5% and specificity of 93.6% with a cut-off value of 2.75. Additionally neuropathy prediction ability of section B is found to be higher than section A. Increasing the score of section B increases the risk of neuropathy by approximately 4.4-fold when compared to section A. The logistic regression model including both sections of the MNSI-TR explained the presence of neuropathy by the rate of 71.5% accross the nerve conduction test results in diagnosing neuropathy. In the logistic regression analysis conducted by Herman et al., it was found as 27% [17]. Our study shows that the Turkish version of the MNSI was effective in the detection of distal peripheral neuropathy. Language validity was assessed by content validity tests done by seven experts separately for each item and the total scale. Our results demonstrated that I-CVI values were between 0.85 and 1 and the S-CVI was 0.95. Expert agreement of at least 0.80 for the scale items is recommended [24,27]. In this study, both I-CVI and S-CVI were over 0.80, indicating that the MNSI-TR maintains the integrity of the original. High agreement between experts demonstrates that the entire instrument and its individual items were appropriate.

### 4.1. Reliability

The test-retest method is commonly used to evaluate the reliability of a scale, which is assessed with test-retest analysis. In this method, when the instrument is applied to the same individual at different times, there should be no difference between the measurements and the correlation should be >0.70 for instrument stability [27]. The test-retest ICC values of the Portuguese and Brazilian Portuguese versions of the MNSI were 0.91 and 0.864, respectively [18,19]. In the present study, the test-retest analysis showed no difference between the measurements and the correlation between the two measurements was 0.97. Being high correlation between the test-retest results of the MNSI-TR, indicating high reliability as with the other language versions. We also used ICC values for the participants’ responses to evaluate the reliability of the whole instrument. The ICC should be >0.60 and as close to 1 as possible [25,26]. The ICC value obtained in our study was greater than 0.60 for section A, indicating that the Turkish version of the questionnaire is adequately reliable. The lower ICC value for section B (ICC:0.439) may be explained by the fact that it was affected by the different clinical attributes of each patient and section B was performed by the clinician.We evaluated the reliability of the Turkish version of the MNSI using KR-20 coefficient, item-total score correlations, and floor/ceiling effect. A KR-20 coefficient over 0.60 is recommended to show that the items have integrity and the test is homogeneous [25,26]. In the present study, KR-20 values were over 0.60 for both sections of the MNSI-TR, indicating high reliability.Another method for assessing reliability is the item-total score analysis. The correlation between the items and total score were determined in this study. Item-total score correlation analysis assesses the conformity of the items to the general structure of the questionnaire, which is analyzed separately for each item. The item-total score correlation should be greater than 0.20 [27,28] and it is recommended to exclude items with correlation coefficients less than 0.20. In our study, all items except the 7, 13, and 15 were sufficiently correlated. Normally, these items should have been removed from the Turkish questionnaire due to their low correlation coefficient. However, according to Herman et al. and the Diabetes Control and Complications Trial/Epidemiology of Diabetes Interventions and Complications (DCCT/EDIC) Research Group, the sensitivities of items 7, 13, and 15 were 14.6%, 11.0%, and 4.2% and their specificities were 94.4%, 90.9%, and 99.5%, respectively [17]. Although their sensitivity is low, their high specificity values indicated that these items can exclude the presence of diabetic neuropathy; thus, it was deemed appropriate not to remove these three items from the MNSI-TR. In addition, questions 4 and 10 in section A were not included in the Turkish version, similar to the original instrument by Feldman et al. [14]. However, after analyzing the answers to questions 4 and 10, we noted that their correlation coefficients were greater than 0.20, which shows a strong association with the integrity of the instrument. In the updated version of MNSI by Herman et al. and DCCT/EDIC, questions 4 and 10 were reintegrated into the instrument due to their high sensitivity, especially question 4 [17]. Our results were also consistent with the updated version of MNSI, although we remained loyal to the original MNSI. However, in the future, we believe that an updated version of the Turkish MNSI should be studied with a much larger patient group. Since the correlation coefficient for each item in section B was above 0.20, it was considered to be consistent with the general structure of the instrument. Tables 3 and 5 show the correlations between the items and the total score found in this analysis.Determining floor and ceiling effects are also recommended as an indicator of scale reliability. Minimum score obtained from the scale gives the floor effect while the maximum score gives the ceiling effect. If the proportion of respondents receiving floor and ceiling scores exceeds 15%, the scale may pose a problem both in reliability and validity. Higher values of the floor and ceiling effect indicate that the responses are stacked, the homogeneous structure deteriorated, and the internal consistency reduced [25,27]. We determined floor and ceiling effects below 15% for both section A and B. However, in the original study and some of the other language adaptation studies, item-total score correlation and ceiling/floor effect evaluations were not performed. Briefly, our results demonstrate that both sections of the MNSI-TR can detect the desired characteristics and have adequate item reliability.

### 4.2. Validity

In ROC curve analysis, sensitivity refers to the power of a test to identify those who are truly ill while specificity shows the test’s ability to identify the truly healthy. For section A of the MNSI-TR, a cut-off point of 3.5 yielded a sensitivity of 75.5% and specificity of 68.1%. When scoring section A of the MNSI, each item receives 0 or 1 point; therefore, because all scores were whole numbers, we determined the minimum point as 4 for the detection of neuropathy. In the Portuguese version, the cut-off point for section A was found to be 3 with 100% sensitivity and 64% specificity [18]. In the original study, Feldman et al. identified a cut-off point of 7 for section A [14]. Herman et al. and the DCCT/EDIC Research Group observed that the cut-off point of 7 had caused many neuropathy cases to be overlooked; therefore, the cut-off point was updated to 4, which had a sensitivity of 0.40 and specificity of 0.92 [17]. This cut-off point was also consistent with our study.The cut-off point for section B of the MNSI was determined as 2.75, which yielded a sensitivity of 87.5% and specificity of 93.6% (Figure 2) However, as each item in section B is worth 0.5 points, the next best minimum point for the detection of neuropathy was 2.50. In the Portuguese version, the cut-off point of section B was 2 with a sensitivity of 100% and a specificity of 86% [18]. Another validation study indicated a cut-off point of 2 with a sensitivity of 65 % and specificity of 83% [16]. In a study involving an ambulatory screening of peripheral neuropathy, the cut-off point was determined as 2.5 with a sensitivity of 78.6% and specificity of 78.6% [15]. In the original study, the cut-off point was 2 with a sensitivity of 80% and a specificity of 95%, while in the updated version (Herman et al. and DCCT/EDIC) the cut-off point was determined as 2.5 with the 61% sensitivity and the 97% specificity [14,17]. The cut-off point identified in our study for section B of the MNSI-TR was consistent with that of the updated version of the MNSI.In the updated MNSI, AUC values were defined as 0.75 for section A and 0.76 for section B [17]. In the Portuguese version, the AUC value was found as 0.913 for section A and 0.798 for section B, respectively [18]. In another study, section A was not evaluated but the AUC of section B was 0.815 [16]. In the Turkish version, we determined that the AUC was > 0.70 for section A, consistent with the previous studies (Figure 1). The AUC for section B was >0.90 (Figure 2), suggesting that section B of the MNSI-TR has more power to differentiate neuropathy compared to other studies.A limitation of this study was that we included illiterate patients. However, we assumed that11.8% illiteracy in our patient group would not affect the results because both their relatives and a health professional were present to read and explain while they took the written part of the test (section A).In conclusion, the MNSI-TR with cut-off values of 3.5 for section A and 2.75 for section B is a useful and reliable method for evaluating peripheral neuropathy. However, according to the MNSI scoring system, since each item represents 1 point for section A and 0.5 or 1 point for section B, accepting the minimum score as 4 points for section A and minimum score as 2.5 for section B confirms the presence of neuropathy. The use of MNSI-TR is a practical and useful method for screening neuropathy in Turkish-speaking societies.

## Acknowledgments

We would like to thank everyone whose opinions helped improve our research. The authors have no funding to report.

## Conflict of interest

Authors have no conflict of interest to declare.
